# Healthcare-Associated *Legionella* Disease: A Multi-Year Assessment of Exposure Settings in a National Healthcare System in the United States

**DOI:** 10.3390/microorganisms9020264

**Published:** 2021-01-28

**Authors:** Meredith Ambrose, Gary A. Roselle, Stephen M. Kralovic, Shantini D. Gamage

**Affiliations:** 1 National Infectious Diseases Service, Specialty Care Services, Veterans Health Administration, Department of Veterans Affairs, Washington, DC 20420, USA; Meredith.Ambrose@va.gov (M.A.); Gary.Roselle@va.gov (G.A.R.); Stephen.Kralovic@va.gov (S.M.K.); 2 Division of Infectious Diseases, Department of Internal Medicine, University of Cincinnati College of Medicine, Cincinnati, OH 45267, USA; 3 Medical Service, Cincinnati VA Medical Center, Cincinnati, OH 45220, USA

**Keywords:** Legionnaires’ disease, *Legionella*, healthcare-associated, healthcare exposure

## Abstract

Healthcare facilities are high-risk environments for *Legionella* disease (LD), including Legionnaires’ disease, but transmission in these settings is often overlooked. We used the LD database at the U.S. Department of Veterans Affairs (VA) national healthcare system to assess the type of healthcare exposure for LD cases. Cases were extracted from the database for 1 September 2012 through 31 July 2019, focusing on cases with an overnight stay at a VA facility during the 10-day exposure window prior to symptom onset. Patient medical charts were reviewed for demographics and types of healthcare setting exposure(s). There were 99 LD cases in the cohort: 31.3% were classified as having definite VA exposure, 37.4% were classified as possible VA with inpatient exposure, and 31.3% were classified as possible VA with both inpatient and outpatient exposure. For definite VA LD cases, 67.7% had some type of exposure in the long-term care setting. While 63% of the 99 cases had exposure in the acute care setting only, both the long-term care and acute care settings contributed substantially to the total number of exposure days. A review of patient movement during the exposure period showed the variable and sometimes extensive use of the VA system, and it provides insights useful for epidemiologic investigations and potential preventive actions.

## 1. Introduction 

The water-based bacterium, *Legionella*, causes infections in humans collectively referred to here as *Legionella* disease (LD) [1]. Infections are usually respiratory, the most severe being a type of pneumonia called Legionnaires’ disease [2,3]. *Legionella* bacteria, found naturally in water, can proliferate in building water distribution systems (e.g., hotels, healthcare facilities) [4,5,6] and lead to LD cases or outbreaks [7,8,9]. Individuals at higher risk for LD include those over 50 years in age, smokers, and those with comorbidities [1,10]. Disease caused by *Legionella* is acute and typically occurs 2 to 14 days after exposure. Cases of LD, while likely underdiagnosed [11], have been increasing over time [3,10,12] as have LD-associated hospitalizations [13].

Healthcare-associated LD (HCA LD) occurs when a person with a *Legionella* infection may have been exposed to the bacteria at a healthcare facility. HCA LD cases have been traditionally classified into categories of either definite or possible based on the extent of healthcare exposure [14]. Per the United States (U.S.) Council on State and Territorial Epidemiologists [15] and adopted by the U.S. Centers for Disease Control and Prevention (CDC) [16], prior to 2020, a case was classified as definite HCA LD if the person had continuous stay in a healthcare facility greater than or equal to 10 days immediately prior to symptom onset. Classification of an LD case as possible HCA LD prior to 2020 occurred when the patient had some exposure to a healthcare facility in the 10 days prior to symptom onset [15]. These definitions have since been updated to account for an expanded exposure window of 14 days prior to LD symptom onset [17,18]. The definitions are broad for healthcare settings and, in addition to hospitals, they include exposure to settings such as long-term care facilities and outpatient clinics. 

Despite the standardized definitions for HCA LD in the U.S., there is little information available about the contribution of the types of exposure to a healthcare setting and occurrence of definite and possible HCA LD, especially for sporadic cases outside the setting of an outbreak. A review of 553 HCA LD cases in 21 US jurisdictions found that 3% of cases were definite HCA LD and 17% were possible HCA LD [8]. Rates of 3% and 30% for definite and possible healthcare association, respectively, were reported in the Department of Veterans Affairs (VA) healthcare system, with most possible HCA LD cases having outpatient exposure only [14]. Both papers noted long-term care being substantially associated with definite HCA LD, but detailed reporting of healthcare setting exposures was not included. 

The VA Veterans Health Administration (VHA) is well poised to fill this gap given the availability of a centralized LD reporting system and a national electronic health record (EHR). In addition, the VHA operates the largest integrated healthcare system in the U.S., and it offers a spectrum of care such as inpatient, long-term care, outpatient, rehabilitation, mental health, and spinal cord injury (SCI) care at 170 medical facilities and 1074 outpatient clinic located across the U.S. and its territories [19]. As of Federal Fiscal Year 2018, the VHA served approximately 6.3 million veterans across its healthcare facilities [19,20]. Using multiple years of HCA LD and EHR data in the VHA system, we examined potential sources of exposure, focusing on those cases with overnight stays in VHA facilities. We demonstrate the complexity of associating healthcare utilization with exposure source in many cases, reinforcing the need to consider thorough epidemiological investigations for all HCA LD cases with overnight stays.

## 2. Materials and Methods 

The VHA centrally collects LD case data from VHA medical facilities for laboratory-confirmed community- and VA-associated cases (see the Supplemental Content in Reference 14 for a listing of data elements in the LD Case Report database). We extracted LD cases from the LD Case Report database for 1 September 2012, through 31 July 2019. During this time period, cases were reported by VHA medical facilities, using VA definitions based on CDC definitions, as non-VA LD, definite VA LD, or possible VA LD (Table 1).

The VHA *Legionella* prevention policy includes the requirement for routine quarterly testing of healthcare buildings for *Legionella*, even in the absence of cases, to validate primary prevention efforts. Therefore, also captured in the LD Case Report database is whether the person had contact with a VA building that tested positive for *Legionella* during the cycle of routine water testing immediately prior to the occurrence of the case (see the Supplemental Content in Reference [14] for the VA water testing policy).

To identify patients that experienced an overnight stay at a VA facility in the 10 days prior to symptom onset, the extracted dataset was filtered to remove patients classified as non-VA LD or possible-outpatient VA LD. For the remaining patients with a reported classification of definite, possible-inpatient or possible-both VA LD, LD diagnosis and case classification were confirmed through a review of the patients’ medical charts in the VHA EHR. Each verified patient was given a unique identification number for tracking purposes. Medical charts for these patients were also reviewed for information about demographics, smoking history, intensive care unit (ICU) admission within 30 days of symptom onset, and each patient’s exposure to, and specific location in, the VA healthcare system in the 10 days prior to symptom onset. Locations of potential exposure were collapsed into the following categories: acute care, ICU, community living centers (CLCs; the VHA term for nursing home care units), SCI, domiciliary (i.e., resident treatment programs) and outpatient clinics. 

## 3. Results

**Figure 1:**
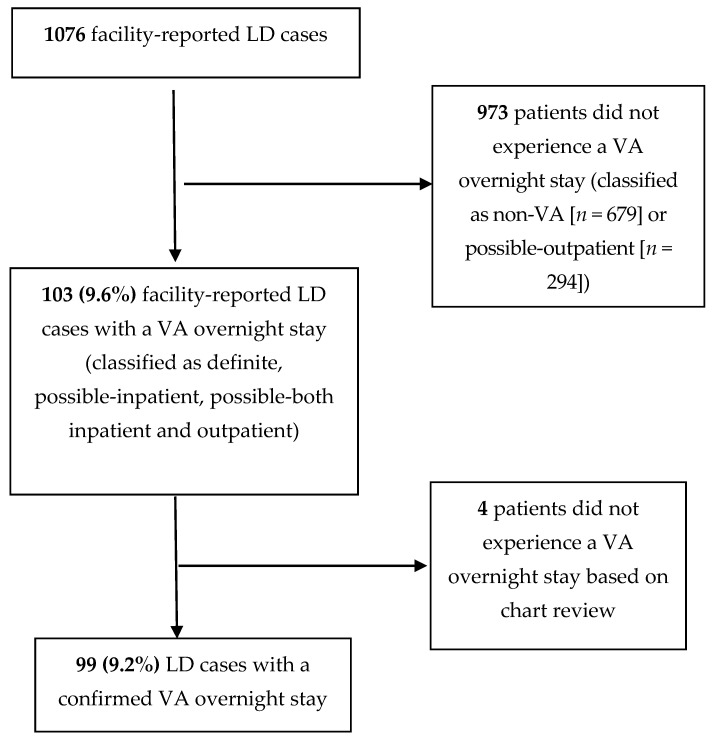
Study population selection from patients entered into the VA LD Case Report database between September 2012 through July 2019 (Abbreviations: VA, Department of Veterans Affairs; LD, *Legionella* Disease).

The initial search of the VHA LD Case Report database yielded 1076 facility-reported LD cases; 973 (90.4%) cases were excluded because they were classified as non-VA LD (679/1076, 63.1%) or classified as possible-outpatient VA LD (294/1076, 27.3%), leaving 103 cases with overnight stays in the 10 days prior to symptom onset for further review (Figure 1). After detailed reviews of patients’ medical charts, four additional cases were excluded because there was no evidence of an overnight stay during the time period of review.

Most LD cases were diagnosed by urinary antigen testing (92.9%). The patient cohort median age was 69 years old, and primarily male (98%), white (64.7%), and a reported current or former smoker (65.7%) at time of symptom onset (Table 2). Of the 99 patients in the cohort, 83 (83.8%) had reported signs or symptoms of pneumonia, 46 (46.5%) had an ICU admission within 30 days of LD symptom onset, and 20 (20.2%) died within 30 days of symptom onset. 

LD cases were reviewed for their epidemiologic classification based on exposure to a VA building in the 10 days prior to symptom onset (Table 3). For the 99 patients, 31 (31.3%) were classified as definite VA LD (2.9% of the full 1076 cases), 37 (37.4%) were classified as possible-inpatient VA LD (3.4% of 1076 cases), and 31 (31.3%) were classified as possible-both VA LD (2.9% of 1076 cases). See Appendix A for percentages of each LD category (Table A1).

Analyzing those cases with VA overnight stays, 14 of 31 (45.2%) definite VA LD cases, 19 of 37 (51.4%) possible-inpatient VA LD cases, and 13 of 31 (41.9%) possible-both VA LD cases had an ICU admission within 30 days. The unadjusted 30-day death rate for the overall period of review was 19.4% (6/31) for definite VA LD, 21.6% (8/37) for possible-inpatient VA LD, and 19.4% (6/31) for possible-both VA LD cases. 

The breakdown of case classifications by calendar year showed that 2017 not only had the highest number of cases overall but also the most definite VA LD cases (Table 3). Most of the VA LD cases in this review were found in the South Atlantic (21.2%), East North Central (19.2%), and Middle Atlantic (15.2%) divisions, with the South Atlantic division accounting for 35.5% of definite VA LD cases (Table 4). These three divisions also had the greatest number of medical facilities contributing to the total LD cases (Table 4). For all divisions, 56 medical facilities contributed to the 99 VA LD cases, with 30 facilities having one case, 13 facilities having two cases, 9 facilities having three cases, and 4 facilities having four cases over the duration of the review. 

**Figure 2:**
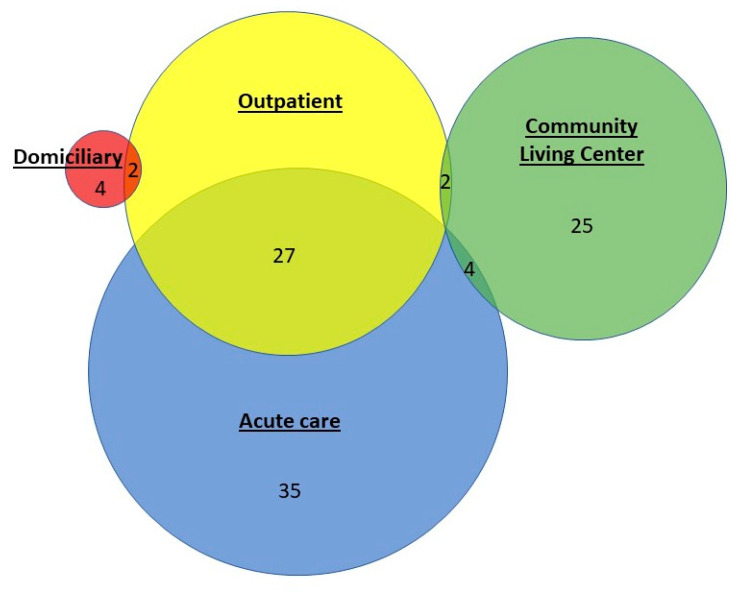
Number of confirmed cases of healthcare-associated *Legionella* disease by VA healthcare setting exposure. For this depiction, the section of the diagram with only acute care exposure (n = 35) includes four cases that had spinal cord injury unit contact only.

To understand the types of healthcare exposure that the patients in this cohort had prior to LD symptom onset, we categorized each patient’s location(s) for the 10-day exposure window using data abstracted from the EHR. A majority of the cases had overnight stays only in the acute care setting (62/99, 63%) followed by only the long-term care setting (27/99, 27.3%); four cases had both acute care and long-term care exposure (Figure 2). There was overlap in the types of settings encountered, with the majority being outpatient visits for those who also had overnight stays in acute care.

**Figure 3:**
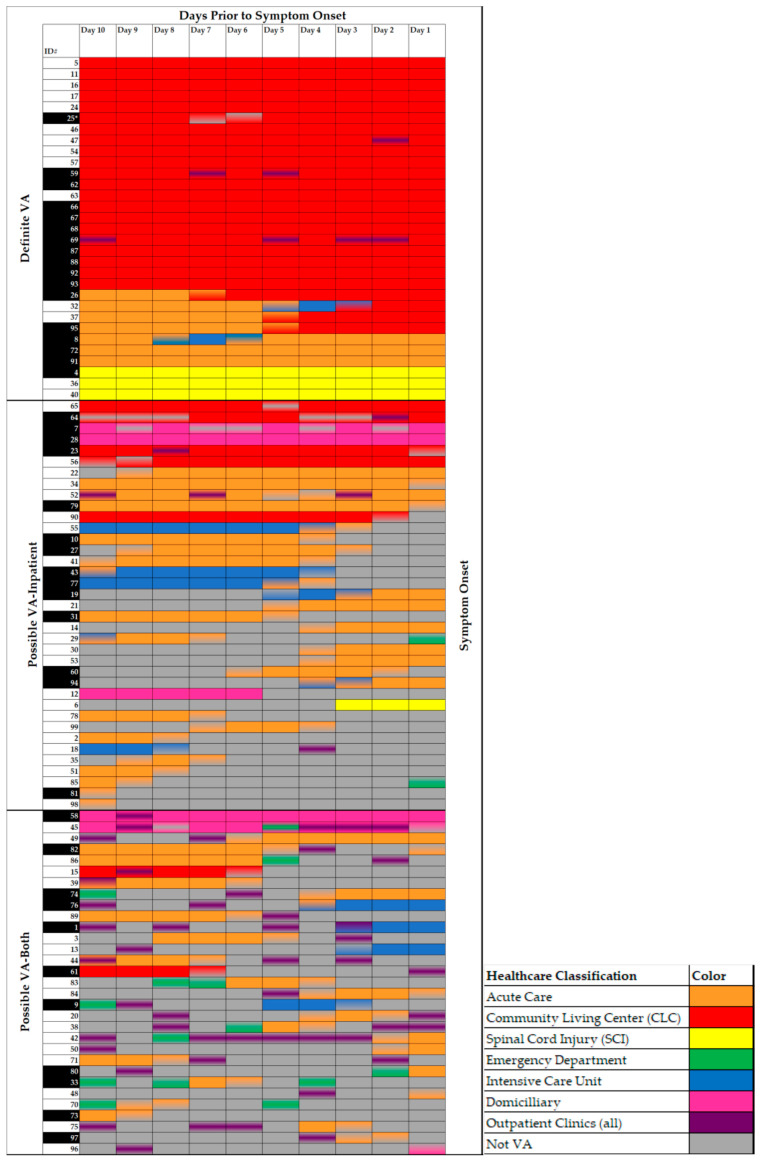
Location of healthcare-associated *Legionella* disease (LD) cases in the 10 days prior to symptom onset. Day 10 represents the first day at the beginning of the review period to determine whether or not the patient had exposure to the VA healthcare system. Patient movement within the healthcare system is depicted by showing clinic appointments as shading (purple) in the middle of the day and admission and discharge is shown as half-shaded days, allowing for visualization of all the points of the healthcare system the patient contacted during the 10-day period. Cases are arranged by classification type. Possible VA LD cases are arranged based on number of days of overnight stay exposure from highest to lowest. The asterisk for case 25 denotes a molecular match between clinical and environmental samples, resulting in the case being classified as definite VA LD despite the person leaving the CLC within the 10-day exposure window. Cases with the ID# shaded black with white writing denote LD case report forms that indicated the patient or resident had contact with a building that tested positive for *Legionella* during the quarterly water testing immediately prior to the occurrence of the case.

We investigated the types of healthcare setting exposures for each patient in more detail, which were categorized by VA LD case classification (Figure 3). Of the 31 definite VA LD cases, 21 (67.7%) had exposure only in a CLC, 3 (9.7%) had exposure only in a SCI unit, 3 (9.7%) had acute care exposure only, and 4 (12.9%) had both acute care and CLC exposure. The majority of patients with possible VA LD exposure (inpatient-only and both inpatient and outpatient) had overnight exposure in acute care in the 10 days prior to symptom onset: 29/68 (42.6%) cases with acute care exposure only and 27/68 (39.7%) with acute care and outpatient exposure.

Figure 3 shows that for definite VA LD cases, there was no to little movement of the patients outside of the CLC or SCI setting in the 10 days prior to symptom onset, and even the few of those with movement transitioned directly from acute to residential settings. However, patient location was much more variable for possible VA LD cases. Twenty-six possible VA LD cases (38%) had at least five overnight stays at a VA facility within the 10-day exposure window. This includes five patients with Domiciliary exposure where the patient was at the setting for five or more overnight stays but had the ability to leave the medical campus during the day. When looking at all 990 exposure days prior to symptom onset (99 cases with 10 days of potential exposure each), most of the exposure was in the CLC setting (282 days, 28.4%) followed by the acute care and ICU settings (244 days, 24.6%).

Forty-two of the 99 cases (42.4%) were reported to have had contact with a VA building that tested positive for *Legionella* in the cycle of routine water testing immediately prior to the occurrence of the case: 17/31 (54.8%) definite VA LD cases, 14/37 (37.8%) possible-inpatient VA LD cases, and 11/31 (35.5%) possible-both VA LD cases (Figure 3). 

## 4. Discussion

This work adds to the limited published surveillance data on HCA LD. Rates of HCA LD and of the specific HCA LD case classifications in this cohort were comparable to previous reports [8,14,16,21], especially for definite HCA LD (2.9% of all LD cases), which arguably is the classification most likely to be determined at facilities based on the longer contact of the person with a healthcare setting and the more obvious need for a case investigation. Comparison of case rates for possible HCA LD in this work (about 30%) with other reports (typically about 15–20%) is more difficult because of a variability in focus on the identification of such cases, particularly those with only outpatient contact. In the rigorous VA LD surveillance system to which VA medical facilities are required to report LD cases [14], one clinic visit in the 10-day exposure window would result in a case being classified and reported as a possible VA LD case. These possible-outpatient VA LD cases accounted for most of the possible VA LD (81.2%) cases. 

The demographics and smoking status of the cases in our cohort were also largely consistent with published reports for LD [10] and with the older and predominantly male patient population accessing VA healthcare [22]. The percentage of LD patients in this report who were identified in the EHR as African American/Black was a higher percentage (30.3%) than the percentage of this race in the VA patient population (approximately 15%) [23]. This finding is in alignment with data reported for 2016–2017 by the CDC of a higher LD rate in African Americans/Blacks in the U.S. compared to other races [16]. 

The unadjusted 30-day death rate was similar to previously reported case fatality rates of about 25% for definite HCA LD [8], substantiating that LD can be a severe illness in patients with lengthy contact with acute care settings and in residents of long-term care facilities who may have comorbidities that affect outcomes. However, the fatality rate in the possible HCA LD cases in our cohort was about double the rate in other studies (10%) [8], which may be because we limited our review to possible HCA LD cases with overnight stays and because persons enrolled in the VHA system are older and have more underlying health conditions [24]. 

While exposure to *Legionella* in healthcare settings has long been recognized as a higher risk situation [10,25,26], there are numerous reports in the literature of multiple HCA LD cases at a facility occurring over years prior to the recognition of a problem [27]. Our findings show in detail the types of contact a person can have with various healthcare settings prior to LD symptoms. Although contact with a setting does not necessarily equate to the source of *Legionella* exposure, especially for possible HCA LD cases, understanding the epidemiologic classifications in the context of types of exposure highlights the scope of comprehensive investigations for healthcare facilities and public health authorities. Our data confirmed the significant association of the long-term care setting on definite HCA LD cases in the U.S. Not only is the temporal definition for definite HCA LD easier to apply in settings where persons have lengthy stays, long-term care residents are often more susceptible to LD, and the settings themselves may have conditions that are conducive to *Legionella* growth such as lower hot water temperatures to prevent scald injury [8,25]. Most of the definite VA LD cases were single such cases at a facility. Facilities with definite VA LD cases received extensive outreach from the VA Central Office, including on-site consultative visits as warranted [28]. Our findings also reinforce data from outbreaks [29] indicating the importance of *Legionella* diagnostic testing in long-term care residents with pneumonia. 

Importantly, we characterized in detail the extent and types of contact of possible HCA LD cases within a healthcare system. Since the definition itself includes contact outside the healthcare facility, there is often a propensity to assume that an LD case temporally classified as possible HCA LD was exposed somewhere else. In addition, the CDC recommends fully investigating possible HCA LD cases when two occur within 12 months, with a maintenance of heightened awareness for other cases and consideration of an environmental review after the first case is identified [30,31]. However, our data showed that a substantial number of such cases spent more than half the 10-day exposure window in a healthcare facility, increasing the likelihood that exposure was at the facility and especially obviating the need to wait for another case before implementing a full investigation or preventive actions. Per VA policy, even a single case of possible VA LD is investigated for likelihood of exposure, which may include water testing (for physical characteristics as well as potential presence of *Legionella*), as a cautionary practice to amplify the prevention of additional cases. Indeed, most of the VA medical facilities with VA LD cases with overnight stays had only one or two such cases in the 7-year review period. For healthcare facilities that do not have such a policy for assessing single cases of possible HCA LD, our data reinforce that the amount of time spent at the facility should be considered and not just the type of case classification for implementing an investigation. 

This review is the first look at environmental *Legionella* data from routine water testing for the validation of a primary prevention program in the context of HCA LD cases over multiple years. More LD cases that were definitely associated with a VA medical facility had *Legionella*-positive water in the recent past compared to cases that were possibly associated with a VA facility, although we note that many cases for both classifications occurred in facilities with *Legionella* not detected in water in the recent past. A number of the definite VA LD cases with past positive water were at long-term care facilities, reinforcing the challenges with maintaining water systems to prevent *Legionella* growth at these settings. Since VA policy requires facilities to have heightened awareness for LD cases if routine water testing detects *Legionella*, our findings need to be interpreted with care because the increased number of definite HCA LD cases in facilities with *Legionella*-positive water could have been the result of more diagnostic LD testing occurring in long-term care buildings with recent positive water results. Furthermore, using these environmental data to imply specific source attributions for the LD cases is not necessarily appropriate, since the testing was not done for case investigations. Therefore, these environmental data show that routine water testing for pathogens is best used to trend data to understand the implementation of engineering controls and not as absolute indications of risk. Future work will review environmental *Legionella* data on a national level in the VA system, including data from facilities that did not have VA LD cases, to better understand trending, risk assessments, and correlations with clinical data. 

This study had limitations. First, there were 973 LD cases that did not meet the initial inclusion criteria because they were facility-reported as either non-VA LD or possible VA LD with only outpatient contact. As a result of the volume of patients, medical charts from those 973 cases were not reviewed to determine if the cases were classified correctly as non-VA or possible VA-outpatient only cases when reported to the VA LD Case Report database, and there could be some cases that should have been included in this review. Second, LD is underdiagnosed in the U.S. [11], since specific testing must be performed to identify cases; it is likely that some cases of LD in the VA system were missed as a result of lack of testing and therefore not available for inclusion in this review. However, as we previously reported [14], VHA performs extensive LD testing as part of its policy to prevent HCA LD [32], which may reduce the likelihood of missed cases compared to other healthcare systems in general. Third, the urinary antigen test was used predominantly to diagnose LD cases, which identifies disease caused by *L. pneumophila* serogroup 1; therefore, cases could have been missed if clinical culture was not performed to diagnose LD caused by other species of *Legionella*. Fourth, the utilization of healthcare may be different by Veterans enrolled in the VA healthcare system than healthcare utilization by the U.S. population based on several factors including increased median age, health status, and access to care, potentially affecting the generalizability of our findings. However, this would result in an overreporting of healthcare contact prior to LD, not underreporting. The findings here can serve as information from a public, widely used system for use by healthcare facilities of any size when assessing the scope of how patients and residents interact with healthcare settings. 

## 5. Conclusions

This retrospective analysis of HCA LD cases in VHA offers a unique perspective on healthcare contact in the LD exposure window from a large healthcare system that has prioritized policies for the prevention and diagnosis of LD cases. There are several findings and recommendations from this work that can inform other healthcare systems: (1) Have a high index of suspicion for LD in patients or residents with HCA pneumonia and perform LD diagnostic testing; (2) conduct case investigations for possible HCA LD cases, especially when the number of days of exposure is higher, to identify potential water system issues and prevent additional HCA LD cases; and (3) residential healthcare settings must prioritize prevention plans to reduce the risk of HCA LD in a vulnerable population. This work also reinforced the need to further explore ways to utilize routine *Legionella* testing data to understand risk and policy effectiveness, but even this preliminary assessment shows the value that collecting such data can have. In summary, healthcare systems can have a wealth of data and information for HCA LD prevention if such programs are prioritized. 

## Figures and Tables

**Table 1:** VA *Legionella* disease case classification categories and definitions used for reporting HCA LD cases ^a^ by VHA medical facilities for cases prior to 2020.

CSTE/CDC Categories and Definitions for HCA LD Cases	VA Categories and Definitions for HCA LD Cases	VHA Medical Facility Settings Where VA Category Could Be Met
**Definite HCA LD:** Patient hospitalized or a resident of a long-term care facility for the entire 10 days prior to onset.	**Definite VA LD:** Patient had *continuous* contact with any VA facility for 10 days prior to symptom onset.	Acute care, nursing home care (called “Community Living Centers”, CLCs), SCI care, and residential programs (called Domiciliaries)
**Possible HCA LD:** Patient had exposure to a healthcare facility for a portion of the 10 days prior to onset	**Possible VA LD:** Patient had *some* contact with any VA facility for 10 days prior to symptom onset. VA further categorizes possible VA LD cases based on type of exposure:	See below for settings based on sub-categories
	**Possible-inpatient VA LD:** Inpatient exposure only for a portion of the 10 days prior to symptom onset	Any setting (not just acute care) where overnight stay is possible; (see list of settings above for Definite VA LD)
	**Possible-outpatient VA LD:** Outpatient or non-clinical exposure only in the 10 days prior to symptom onset	Any contact without an overnight stay. This includes any outpatient clinics such as, but not limited to, dialysis and dental clinics. It also includes non-clinical contact such as picking up a prescription or attending social activities such as bingo.
	**Possible-both VA LD:** Both inpatient and outpatient exposure prior to symptom onset	Includes the settings listed above for Possible-inpatient VA LD *and* Possible-outpatient VA LD. A person must have both types of exposure for the case to be categorize as Possible-both.

Abbreviations. CSTE, Council of State and Territorial Epidemiologists; CDC, Centers for Disease Control and Prevention; HCA, Healthcare-associated; LD, *Legionella* Disease; SCI, spinal cord injury; VA, Department of Veterans Affairs; VHA, Veterans Health Administration. ^a^ Cases with no exposure to a VHA medical facility in the 10 days prior to symptom onset were classified as non-VA LD.

**Table 2:** Demographics, smoking status, and Legionella disease indicators of 99 HCA LD patients.

Characteristic	All Patients (*n* = 99)	Percent
**Demographic information**		
**Age**		
30–39	1	1.0
40–49	2	2.0
50–59	10	10.1
60–69	43	43.4
70–79	25	25.3
80–89	17	17.2
90+	1	1.0
**Sex**		
Female	2	2.0
Male	97	98.0
**Race**		
African American/Black	30	30.3
American Indian/Alaska Native	1	1.0
Asian	1	1.0
Native Hawaiian/Pacific Islander	1	2.0
White	64	64.7
Not stated	2	2.0
**Ethnicity**		
Hispanic	7	7.1
Non-Hispanic	89	89.9
Not stated	3	3.0
**Smoking Status at LD Symptom Onset ^a^**		
Current	34	34.3
Former	31	31.3
Never	33	33.3
Unknown	1	1.0
		
***Legionella*** **disease information**		
**Pneumonia ^b^**		
No	16	16.2
Yes	83	83.8
**Death within 30 days of Symptom Onset**		
No	79	79.8
Yes	20	20.2
**ICU Admission within 30 days** **of Symptom Onset**		
No	53	53.5
Yes	46	46.5

Abbreviations. HCA, healthcare-associated; LD, *Legionella* Diseases; ICU, intensive care unit. ^a^ Smoking status was determined by reviewing the “History and Physical” or “Admission Assessment” in patient medical charts around the time of LD symptom onset. Based on this assessment, smoking status was categorized as current, former (previously smoked and quit regardless of when), never, or unknown. ^b^ Presence of pneumonia for each LD case was facility-reported as part of the central collection of LD data. Facilities are asked whether the patient had radiologic evidence and/or displayed signs/symptoms of pneumonia.

**Table 3:** VA LD cases, by case classifications and calendar year of diagnosis.

Diagnosis Year ^a^	Definite	Possible-Inpatient	Possible-Both	Total
2012	0	0	0	**0**
2013	1	1	2	**4**
2014	9	6	5	**20**
2015	5	5	5	**15**
2016	1	2	5	**8**
2017	11	8	7	**26**
2018	3	10	6	**19**
2019	1	5	1	**7**
**Total**	**31**	**37**	**31**	**99**

^a^ LD cases in 2012, 2013, and 2014 (up to 15 October 2014) were retrospectively reported to the LD Case Report database by facilities; after 15 October 2014, LD cases were reported as they occurred. No cases from 1 September 2012 (the start of the data review period) to the end of that year fit the inclusion criteria. Cases for 2019 are included up to 1 July 2019, the end of the review period.

**Table 4:** VA LD cases, by case classifications and U.S. Census Division.^a.^

US Division (No. Medical Facilities) ^b^	Definite	Possible-Inpatient	Possible-Both	Total
New England (3)	1	3	2	**6**
Middle Atlantic (10)	3	6	6	**15**
East North Central (11)	6	9	4	**19**
West North Central (5)	0	3	4	**7**
South Atlantic (11)	11	5	5	**21**
East South Central (2)	3	1	1	**5**
West South Central (5)	4	2	3	**9**
Mountain (4)	1	4	2	**7**
Pacific (5)	2	4	4	**10**
**Total**	**31**	**37**	**31**	**99**

^a^ 2010 U.S. Census divisions available at: https://www2.census.gov/geo/pdfs/maps-data/maps/reference/us_regdiv.pdf. Since Puerto Rico is not included in the Census divisions, any LD cases at the VA medical facility in Puerto Rico were included in the South Atlantic division to align with other VA medical facilities in the same VA region. ^b^ Number of VA medical facilities that contributed to the total number of LD cases for each division.

## Data Availability

The data presented in this work are not publicly available because they contain sensitive, protected information about individuals that may be identifiable. Contact the correcsponding author for data requests, which will need to meet all criteria in Department of Veterans Affairs policies for the sharing of patient information before approval can be granted.

## Abbreviations

CDC	Centers for Disease Control and Prevention
CLC	community living center
CSTE	Council of State and Territorial Epidemiologists
EHR	electronic health record
HCA	healthcare-associated
ICU	intensive care unit
LD	Legionella disease
SCI	spinal cord injury
US	United States
VA	US Department of Veterans Affairs
VHA	Veterans Health Administration

## Appendix A

*Legionella* disease (LD) cases were categorized for VA association using the definitions in Table 1, and percentages of each category were assessed based on all LD cases in the review period and only those VA LD cases with VA overnight stay exposure (Table A1).

**Table A1:** LD case classifications by VA healthcare exposure.

LD Case Category ^a^	Number of LD Cases ^b^	% of All LD Cases (*n* = 1076)	% of VA LD Cases with Any VA Overnight Stay Exposure (*n* = 99)
Non-VA LD	679	63.1%	N/A
Definite VA LD	31	2.9%	31.3%
Possible VA LD	362	33.6%	
**Sub-categories of Possible-VA LD**			
Possible-outpatient VA LD	294	27.3%	N/A
Possible-inpatient VA LD	37	3.4%	37.4%
Possible-both VA LD	31	2.9%	31.3%
Possible-inpatient *and* Possible-both VA LD	68	6.3%	68.7%

Abbreviations: LD, *Legionella* disease; VA, Department of Veterans Affairs; N/A, not applicable. ^a^ See Table 1 for LD case category definitions. ^b^ Excludes 4 cases that were reported as having VA overnight stays, but no overnight stays were found in medical chart reviews.

## References

[1] Fields B.S., Benson R.F., Besser R.E. (2002). Legionella and Legionnaires’ Disease: 25 Years of Investigation. Clin. Microbiol. Rev..

[2] McDade J.E., Shepard C.C., Fraser D.W., Tsai T.R., Redus M.A., Dowdle W.R., The Laboratory Investigation Team (1977). Legionnaires’ Disease: Isolation of a Bacterium and Demonstration of its Role in Other Respiratory Diseease. N. Engl. J. Med..

[3] Hicks L.A., Garrison L.E., Nelson G.E., Hampton L.M. (2011). Legionellosis in the United States, 2000–2009. Morb. Mortal. Wkly. Rep..

[4] Leoni E., De Luca G., Legnani P., Sacchetti R., Stampi S., Zanetti F. (2004). Legionella waterline colonization: Detection of Legionella species in domestic, hotel and hospital hot water systems. J. Appl. Microbiol..

[5] Sikora A., Wójtowicz-Bobin M., Kozioł-Montewka M., Magryś A., Gładysz I. (2015). Prevalence of Legionella pneumophila in water distribution systems in hospitals and public buildings of the Lublin region of eastern Poland. Ann. Agric. Environ. Med..

[6] Bédard E., Paranjape K., Lalancette C., Villion M., Quach C., Laferrière C., Faucher S.P., Prévost M. (2019). Legionella pneumophila levels and sequence-type distribution in hospital hot water samples from faucets to connecting pipes. Water Res..

[7] Garrison L.E., Kunz J.M., Cooley L.A., Moore M.R., Lucas C., Schrag S., Sarisky J., Whitney C.G. (2016). Vital Signs: Deficiencies in Environmental Control Identified in Outbreaks of Legionnaires’ Disease—North America, 2000–2014. Mmwr. Morb. Mortal. Wkly. Rep..

[8] Soda E.A., Barskey A.E., Shah P.P., Schrag S., Whitney C.G., Arduino M.J., Reddy S.C., Kunz J.M., Hunter C.M., Raphael B.H. (2017). Vital Signs: Health Care–Associated Legionnaires’ Disease Surveillance Data from 20 States and a Large Metropolitan Area—United States, 2015. Mmwr. Morb. Mortal. Wkly. Rep..

[9] Watkins L.K.F., Toews K.-A.E., Harris A.M., Davidson S., Ayers-Millsap S., Lucas C.E., Hubbard B.C., Kozak-Muiznieks N.A., Khan E., Kutty P.K. (2017). Lessons From an Outbreak of Legionnaires’ Disease on a Hematology-Oncology Unit. Infect. Control. Hosp. Epidemiol..

[10] National Academies of Sciences, Engineering, and Medicine (2019). Management of Legionella in Water Systems.

[11] Collier S.A., Deng L., Adam E.A., Benedict K.M., Beshearse E.M., Blackstock A.J., Bruce B.B., Derado G., Edens C., Fullerton K.E. (2021). Estimate of Burden and Direct Healthcare Cost of Infectious Waterborne Disease in the United States. Emerg. Infect. Dis..

[12] Centers for Disease Control and Prevention Nationally Notifiable Infectious Diseases and Conditions, United States: Annual Tables, 2018. https://wonder.cdc.gov/nndss/static/2018/annual/2018-table2h.html.

[13] Mudali G., Kilgore P.E., Salim A., McElmurry S.P., Zervos M. (2020). Trends in Legionnaires’ Disease-Associated Hospitalizations, United States, 2006–2010. Open Forum Infect. Dis..

[14] Gamage S.D., Ambrose M., Kralovic S.M., Simbartl L.A., Roselle G.A. (2018). Legionnaires Disease Surveillance in US Department of Veterans Affairs Medical Facilities and Assessment of Health Care Facility Association. JAMA Netw. Open.

[15] Council of State and Territorial Epidemiologists Public health reporting and national notification for legionellosis. Position statement no. 09-ID-45. Atlanta, GA: Council of State and Territorial Epidemiologists; 2009. https://c.ymcdn.com/sites/w]ww.cste.org/resource/resmgr/PS/09-ID-45.pdf.

[16] Centers for Disease Control and Prevention (2020). Legionnaires’ Disease Surveillance Summary Report, 2016–2017. https://www.cdc.gov/legionella/health-depts/surv-reporting/2016-17-surv-report-508.pdf.

[17] Council of State and Territorial Epidemiologists Revision to the Case Definition for National Legionellosis Surveillance. https://cdn.ymaws.com/www.cste.org/resource/resmgr/2019ps/final/19-ID-04_Legionellosis_final.pdf.

[18] Centers for Disease Control and Prevention Legionellosis Case Report form. January 2020. https://www.cdc.gov/legionella/downloads/case-report-form.pdf.

[19] Department of Veterans Affairs Veterans Health Administration. https://www.va.gov/health/aboutVHA.asp.

[20] Department of Veterans Affairs VA Benefits & Healthcare Utilization Pocket Cards. https://www.va.gov/vetdata/docs/pocketcards/fy2019q4.pdf.

[21] Beauté J., Plachouras D., Sandin S., Giesecke J., Sparén P. (2020). Healthcare-Associated Legionnaires’ Disease, Europe, 2008−2017. Emerg. Infect. Dis..

[22] Department of Veterans Affairs National Center for Veterans Analysis and Statistics. Veteran Population.. https://www.va.gov/vetdata/Veteran_Population.asp.

[23] Department of Veterans Affairs National Veteran Health Equity Report-FY 2013. https://www.va.gov/HEALTHEQUITY/docs/National_Veterans_Health_Equity_Report_FY2013_FINAL_508_Comp.pdf#page=19.

[24] Wilson N.J., Kizer K.W. (1997). The VA health care system: An unrecognized national safety net. Heal. Aff..

[25] World Health Organization (2007). Legionella and the Prevention of Legionellosis.

[26] Barker K.A., Whitney E.A., Blake S., Berkelman R.L. (2015). A Review of Guidelines for the Primary Prevention of Legionellosis in Long-Term Care Facilities. J. Am. Med Dir. Assoc..

[27] Gamage S.D., Ambrose M., Kralovic S.M., Roselle G.A. (2016). Water Safety and Legionella in Health Care: Priorities, Policy, and Practice. Infect. Dis. Clin. North Am..

[28] Ambrose M., Kralovic S.M., Roselle G.A., Kowalskyj O., Rizzo V., Wainwright D.L., Gamage S.D. (2020). Implementation of Legionella Prevention Policy in Health Care Facilities: The United States Veterans Health Administration Experience. J. Public Heal. Manag. Pr..

[29] Skaza A., Beskovnik L., Štorman A., Keše D., Ursic S. (2011). Epidemiological investigation of a legionellosis outbreak in a Slovenian nursing home, August 2010. Scand. J. Infect. Dis..

[30] Tablan O.C., Anderson L.J., Besser R., Bridges C., Hajjeh R. (2004). Guidelines for preventing health-care-associated pneumonia. Recommendations of CDC and the Healthcare Infection Control Practices Advisory Committee. Mmwr Morb. Mortal. Wkly Rep..

[31] Centers for Disease Control and Prevention Things to Consider: Healthcare-associated Cases and Outbreaks. https://www.cdc.gov/legionella/health-depts/healthcare-resources/cases-outbreaks.html.

[32] Veterans Health Administration (VHA) Prevention of healthcare-associated Legionella disease and scald injury from potable water distribution system. Washington, DC: VHA Directive 1061; 2014. https://www.va.gov/vhapublications/ViewPublication.asp?pub_ID=3033.

